# Should SVGp12 Be Used for JC Polyomavirus Studies? Comment on Prezioso et al. COS-7 and SVGp12 Cellular Models to Study JCPyV Replication and MicroRNA Expression after Infection with Archetypal and Rearranged-NCCR Viral Strains. *Viruses* 2022, *14*, 2070

**DOI:** 10.3390/v15010089

**Published:** 2022-12-29

**Authors:** Stian Henriksen, Christine Hanssen Rinaldo

**Affiliations:** 1 Department of Microbiology and Infection Control, University Hospital of North Norway, N-9038 Tromsø, Norway; 2 Metabolic and Renal Research Group, Department of Clinical Medicine, UiT The Arctic University of Norway, N-9037 Tromsø, Norway

**Keywords:** polyomavirus, BKPyV, JCPyV, SVGp12, exosomes, microRNA, infection, replication

## Abstract

A recent paper in *Viruses* investigates the impact of the JC polyomavirus (JCPyV) microRNA on the replication of different JCPyV strains. Unfortunately, one of the cell lines used, the human fetal glial cell line SVGp12, is productively infected by the closely related BK polyomavirus (BKPyV), which may confound results. Scientists need to take this into account and the potential pitfalls.

The JC polyomavirus (JCPyV) infects 50% to 90% of humans worldwide and causes lifelong persistent infection of the epithelial cell lining of the renourinary tract, and possibly latent infection of glial cells in the brain and hematopoietic cells in the bone marrow [1]. Although usually not associated with ill effects, JCPyV may cause progressive multifocal leukoencephalopathy (PML), a usually fatal disease mainly affecting immunocompromised patients [1,2]. As there is no effective antiviral treatment available, more research on JCPyV replication is urgently needed. In a recent paper published in *Viruses* [3], JCPyV replication and microRNA (miRNA) expression was compared for two JCPyV variants having archetypal- and rearranged-non-coding control regions, respectively. As cell culture models, the authors used COS-7 (ATCC CRL-1651), an African green monkey kidney fibroblast-like cell line, and SVGp12 (ATCC CRL-8621), a human fetal glial cell line. Both cell lines were originally made by transformation with a replication-defective simian virus 40 (SV40) genome, and express the regulatory protein SV40 Large Tumour antigen (LTag) that supports the replication of JCPyV. The study investigated the intracellular viral load and the expression of the viral capsid protein Vp1 three to 35 days post-infection (d.p.i), the expression of the JCPyV miRNA miR-J1-5p [4] in infected cells and purified exosomes, and the effect of pre-treatment with exosomes before infection. The miRNA miR-J1-5p has previously been found to downregulate the expression of JCPyV LTag [4]. Pretreatment with exosomes significantly reduced JCPyV replication. Although some of their results and conclusions were difficult to interpret, they seem to conclude that miRNA expression was inversely correlated with JCPyV replication and was higher during infection with the archetype variant than with the rearranged variant.

Unfortunately, it seems that the authors were not aware that the SVGp12 cell line is persistently infected by the closely related BK polyomavirus (BKPyV) (Figure 1) [5].

The SVGp12 cell line has previously been used for JCPyV studies (reviewed in [5]) and other studies in which cells of neural origin were desirable. In 2014, we unexpectedly detected that the cell line was productively infected by BKPyV [5]. The evidence was unambiguous. Immunofluorescence staining and immunoblotting showed expression of Vp1, Vp2/Vp3 and agnoprotein. Importantly, the antiserum used for agnoprotein detection was generated for BKPyV agnoprotein and had previously been found to not cross-react with SV40- or JCPyV-agnoprotein (Figure 1B) [5]. In agreement with this, a BKPyV-specific quantitative PCR (qPCR) detected high viral loads in both cell culture supernatants and in cells in original vials from ATCC. Moreover, the supernatants were found to contain infectious BKPyV, and DNA sequencing revealed a mixture of complete BKPyV genomes and deletion derivatives. Of note, some of the above-described experiments were performed in two labs more than 3300 kilometres apart, on vials of SVGp12 independently acquired from ATCC. The BKPyV contamination was confirmed by the group of Dr Major at The National Institute of Health in Bethesda, Maryland, the group that created the SVG cells [6]. They convincingly demonstrated that the contamination must have occurred after the deposition of SVG cells in the ATCC in 1984.

Information about the contamination and reference to our work has been available in the product information from ATCC since 2014. Our findings were also described in a review of in vitro and in vivo models for human polyomavirus infection [7]. Although we only find one additional paper studying JCPyV infection in SVGp12 cells after our publication [8], we find 20 other publications mainly focusing on neuroscience or cancer (https://www.atcc.org/products/crl-8621, accessed on 8 November 2022). The fact that the SVGp12 cell line is still extensively used, indicates that many researchers are not aware that this cell line is producing high levels of infectious BKPyV.

## 1. Could the Persistent BKPyV Infection Have Been Detected with the Methodology Used in the Study of Prezioso et al.?

**Figure 1:**
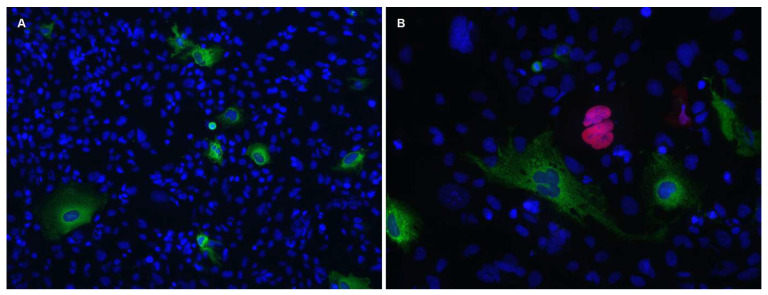
The SVGp12 cell line expresses BKPyV proteins and can be infected by JCPyV. SVGp12 cells were (**A**) mock-infected or (**B**) infected with JCPyV (Mad-4). Cells were fixed 4 d.p.i. and indirect immunofluorescence staining was performed using a BKPyV-specific agnoprotein rabbit polyclonal antiserum in combination with a JCPyV-specific Vp1 mouse monoclonal antibody (ab34756; Abcam). As secondary antibodies goat-anti-rabbit (green, Alexa 488) and goat-anti-mouse (red, Alexa Fluor 568) were used. The DNA (nucleus) was stained with Draq5 (blue). Both images were acquired using a Nikon light microscope with a 10× and 20× objective, respectively.

They used a qPCR to quantitate JCPyV DNA but due to a miss-citation, we do not know the details of this method. The Vp1 expression was investigated by immunoblotting using an antibody that does not cross-react with BKPyV Vp1 (Figure 1) [5], and JCPyV miRNA expression was studied by a qPCR that will not detect BKPyV miRNA [9]. To conclude, with the methods they used, it is unlikely that they could have detected BKPyV.

## 2. In What Way May the Presence of BKPyV Have Affected Their Results?

In Figure 1, we show that only a small subpopulation of SVGp12 cells are infected by BKPyV. We have previously shown that JCPyV infect SVGp12 cells with and without prior BKPyV infection [5]. Prezioso et al [3] did not show immunofluorescence images of their infected cells, but we expect that their infected cultures were quite heterogenous consisting of uninfected-, JCPyV infected-, BKPyV infected- and coinfected cells. A subpopulation of the exosomes, that they purified from these cells, must have originated from BKPyV-infected cells, and hence contained BKPyV miRNA, as well as other components released in response to the BKPyV infection. Importantly, the 3′ miRNA is 100% conserved among BKPyV and JCPyV [10], and therefore, the BKPyV miRNA miR-B1-3p can presumably downregulate JCPyV LTag. Furthermore, during all experiments with SVGp12 cells, BKPyV, proteins and extracellular vesicles released from the persistently infected cells were present, hence adding additional factors with unknown consequences for their results.

## 3. Should SVGp12 Be Used for JCPyV Studies?

The authors of a study from 2016 claimed not to find BKPyV in their SVGp12 cells [8], although their detailed testing was not shown. However, with the indisputable evidence of productive BKPyV infection in at least five ATCC vials of SVGp12 cells, we strongly advise against the use of these cells for JCPyV experiments. Otherwise the cells must first be carefully tested for BKPyV infection. COS-7 cells can be used, but if human cells are preferred, HEK293TT cells [11] or subclones of SVGp12 such as the SVG-A cells [5,6,12] may be used. However, in our experience, COS-7 cells are more permissive for JCPyV [13].
